# The Prognosis in Trench Nephritis

**Published:** 1919

**Authors:** 


**The Prognosis in Trench Nephritis.** By L. C. Dyke. *The Lancet,* September 17, 1918.

Dyke reports 50 after-cases of trench nephritis with a view to throwing light on nephritis.

The mortality was 2% in this series and the cause of death was scarlet fever. In 40% the re-establishment of function was incomplete, and, although not entirely destroyed, the health of the patients was permanently impaired. Susceptibility increases with age and the prognosis becomes worse.

Albuminuria, chronic nephritis, interstitial or parenchymatous, arterio-sclerosis, anemia and edema are the resulting disabilities. Arterio-sclerosis was present in about 50% of patients not entirely restored to health. Search through their past revealed the fact that three of them had had definite attacks of renal trouble previously.

Edema usually disappears during the first week. Its persistence after the second week renders the prognosis increasingly unfavorable.

Albuminuria usually ceases towards the end of the first or during the second month from onset. After three months restoration to complete health is unlikely.

Regarding etiology only two positive deductions were made.

(1) That the incidence among troops is proportionate to the exposure their work entails.

(2) That susceptibility increases with age.

Among suggestions as to the causation of this condition, which have been made in the past, are :

(1) The presence of chloride of lime in the drinking water.

(2) The absence of vitamines from the rations.

(3) Poisoning by heavy metals ingested in solution in the juice of tinned fruit.

(4) The action of poisonous gases.

(5) The action of an already existing infection.

Studies and facts tend to show that none of the above mentioned suggestions are causal agents.

There is one factor, and that is excessive and prolonged fatigue in combination with exposure. It has long been recognized that albumin and casts are almost constantly present in the urine after exertion entailed by athletics. This albuminuria is stated always to clear up. That is the case for athletes who, after the game is over, can take a rest, but conditions are entirely different for soldiers, where fatigue duties are added to the strain of the battle field. Sleep and rest are inadequate both in length and quality and, therefore, what is but a passing symptom for the athlete may, in the soldier, become a morbid process.

Martin Fisher maintains that acute nephritis is due to a toxic acidosis. Osler remarks that " common febrile albuminuria is the first link leading to acute Bright's disease ". May not the same remark apply to the albuminaturia following exertion?

The great frequency of pulmonary complications, particularly bronchitis, is worthy of notice. Tytler and Ryle draw attention to a deposition of fibrin in the walls of the lung alveoli of some cases which came to postmortem. This condition is not found in ordinary nephritis, nor are hemorrhages into the spleen.

In conclusion it may be repeated that the great frequency of " trench " nephritis is due to prolonged fatigue, combined with exposure to cold and wet.

